# Repair of Lower Eyelid Cicatricial Entropion With Midface Lift, Spacer Graft, and Drill-Hole Canthoplasty

**DOI:** 10.1093/asjof/ojaa045

**Published:** 2020-11-11

**Authors:** Mohammed S Alghoul, Jonathan T Bricker

**Affiliations:** 1 Division of Plastic & Reconstructive Surgery, McGaw Medical Center, Feinberg School of Medicine, Northwestern University, Chicago, IL, USA; 2 McGaw Medical Center, Northwestern University, Chicago, IL, USA

## Abstract

Postoperative cicatricial lower lid retraction is a challenging surgical problem that often disfigures the shape of the eye and has functional consequences. Depending on the severity, more than one surgical procedure may be needed to achieve the desired lower lid shape and position given the recurrent nature of scarring. Concepts of scar release, establishing lower lid vertical height, soft tissue replacement, and midcheek support are discussed in this video.

Postoperative lower eyelid retraction can occur after cosmetic blepharoplasty^1^ or reconstructive procedures^2^ including periorbital bone fracture repair. A rotational component may accentuate the condition and worsen the symptoms and cosmetic appearance further. An ectropion or entropion can result from preferential scarring of the anterior and middle lamella or the posterior lamella, respectively. In addition to increased dryness, corneal exposure, and poor cosmesis, an entropion can also result in trichiasis. Treatment typically involves releasing the scar tissue and reestablishing the lid height, position, and support.^3,4^ Cheek support is paramount for restoring and maintaining lower lid position and, therefore, mid cheek lift is critical to maximize the success of the procedure and minimize relapse.^2,4^ A spacer graft is used to replace the missing tissue component, which often is the nidus for scarring, and to provide vertical lid support. Although not needed in all cases, it is usually reserved for severe cases when significant scar formation restricts the upward movement of the lower lid against the globe.^5,6^ Several spacer grafts were described in the literature with no clear advantage of one over the other.^7,8^ In Video 1, we describe the repair of a severe case of lower lid cicatricial entropion in a 46-year-old female patient who had prior periorbital fracture repair. Vertical midface lifting with drill-hole periorbital anchoring as described by Giovanni Botti was employed in addition to the use of a spacer graft.^4^ The patient improved but developed a recurrent milder retraction due to the contracture of the hard palate mucosa spacer graft. Another interposition acellular dermal matrix spacer graft was successful in restoring the full height of the lower eyelid and resulted to complete correction at a 1-year follow-up. Vertical midface lifting with bone anchoring remained stable at 18 months follow-up.

It has been the senior author’s (M.S.A.) experience that cases of severe lower eyelid cicatricial ectropion or entropion usually require more than one procedure to achieve complete resolution. The aim of the first operation is to reestablish mid-face support and to restore the vertical height and position of the lower lid with overcorrection to give some room for secondary contracture to take place.^9^ This usually presents itself as a rotational deformity while maintaining a normal lid height and can be easily repaired with a redo canthopexy or canthoplasty. It also can present with a recurrent retraction that is usually milder than the original presentation due to spacer graft resorption or contracture.^7^ The latter is what happened in this case, which shows a good example of how the reuse of a spacer graft may be necessary in cases with severe lid shortening and tissue loss. It is reminiscent of building and restoring the lower lid height in a block-by-block fashion. The key, however, in the secondary procedure, if deemed necessary, is to limit dissection as much as possible to minimize postoperative scarring the second time around.
